# Assessing the impact of biosecurity practices and animal welfare in small-scale mountain dairy farming

**DOI:** 10.1038/s41598-024-63841-y

**Published:** 2024-06-10

**Authors:** Thomas Zanon, Mousaab Alrhmoun, Matthias Gauly

**Affiliations:** Faculty of Agricultural, Environmental and Food Sciences, Free University of Bolzano, Piazza Università 5, 39100 Bolzano, Italy

**Keywords:** Biosecurity, Animal welfare, Farm profitability, Milk sales, ClassyFarm, Milk quality, Animal physiology, Sustainability

## Abstract

This study estimates the association between the level of biosecurity, animal welfare, milk quality, and economic performance on 2291 mountain dairy farms which largely differs in management and structure from large dairy operations in the lowlands mainly due to climatic and topographic constraints in mountain areas. The dairy industry’s increasing emphasis on biosecurity is crucial for ensuring animal health, productivity, and disease mitigation. Therefore, in the present study the biosecurity and animal welfare status of mountain dairy farms were assessed considering the official welfare protocol for dairy cows of the Italian ClassyFarm system. Our findings reveal a suboptimal adoption of biosecurity measures, attributed to structural limitations in mountain farms and farmers’ awareness gaps. Despite these challenges, the economic significance of biosecurity adoption is evident, emphasizing potential benefits for farm viability and animal health. Conversely, the study indicates a moderate to good welfare status in assessed farms, reflecting farmers’ awareness of the importance of high welfare standards for dairy cows. Improved welfare positively correlates with milk sales and productivity, highlighting the economic advantages of prioritizing animal well-being. Challenges persist, particularly regarding animal housing deficiencies and related consumer concerns about animal welfare in dairy production. Therefore, targeted interventions and educational initiatives are crucial to empower farmers and promote best practices in biosecurity and animal welfare management. However, addressing resultant production cost increases necessitates society’s willingness to pay more for animal-based food, emphasizing the importance of aligning economic incentives with sustainable farming practices.

## Introduction

In recent years, the dairy industry has witnessed a growing emphasis on biosecurity measures as a means to safeguard animal health, enhance productivity, and mitigate the risks associated with disease outbreaks^1,2^. The implementation of effective biosecurity protocols has become a critical component in ensuring the sustainability and profitability of dairy farms worldwide^3,4^. Biosecurity, in the context of animal husbandry, encompasses a range of management practices and preventive measures designed to minimize the introduction and spread of diseases among livestock and therefore reduce the use of drugs as antimicrobials and associated resistances^5,6^. These measures may include, among others, strict hygiene protocols, controlled access to farms, appropriate vaccination programs, and effective disease surveillance^7^. Assessing the biosecurity status on dairy farms requires the utilization of various methods and indicators to capture the multifaceted nature of this complex concept^3,8^. Researchers and industry professionals have developed several approaches to estimate biosecurity levels, each with its strengths and limitations^4^. One commonly employed method is the use of questionnaires or surveys, which involve collecting data directly from farmers regarding their biosecurity practices^9^. These questionnaires typically cover a wide range of topics, such as farm management practices, disease prevention measures, biosecurity infrastructure, and employee training. Another approach involves conducting on-farm assessments, where trained evaluators visit dairy farms and directly observe the physical infrastructure, biosecurity protocols, and animal health management practices in place^10,11^. In addition to surveys and on-farm assessments, some studies utilize quantitative measures, such as disease prevalence or pathogen testing, to estimate the impact of biosecurity practices on animal health outcomes^12,13^. By analysing disease incidence rates or the presence of specific pathogens, researchers can infer the effectiveness of implemented biosecurity measures in reducing the risk of disease transmission within and between farms^14^. Irrespective of the assessment method, previous studies mostly considered small numbers of dairy farms which allow only limited realistic and proper insights for drawing practical conclusions (e.g.^8,15,16^). Furthermore, few studies analyzed the economic impact of applying biosecurity measures (e.g.^17^) and enhanced animal welfare (e.g.^18^) in dairy farming.

In 2018, Italy launched a new integrated system called Classyfarm that combines data obtained from field surveys of animal welfare and farm biosecurity, antimicrobial use and resistance considering data from the electronic prescription system for veterinary drugs, herd data (health status, production and feed) as well as slaughterhouse assessments on animal health (e.g. lung scores) and welfare (e.g. tail lesions in pigs) for surveilling the potential risk of livestock farms for public health^11,19^. Since 2023 the ClassyFarm System is in use for dairy cattle farms. In a previous study, however, Holighaus et al.^20^ showed that the practicability of the welfare protocol for dairy cows used in the ClassyFarm system for small-scale mountain farms was for some sections (especially biosecurity) limited as topographical and structural constraints hamper farms to fulfill many of the requested criteria. In order to motivate farmers for implementing biosecurity measures and animal welfare criteria in respective farms the present study aims to first investigate the interaction between biosecurity measures and productivity (milk yield, milk quality) and secondly to quantify the economic impact for farms when applying such measures. Hereby, the assessment on biosecurity practices and animal welfare was carried out on 2291 mountain dairy farms to gain realistic and practical insights into the interaction between biosecurity and welfare measures with milk performance (quality and yield) and resulting economic performance.

## Results

Descriptive statistics of herd size, biosecurity score, welfare score, production traits and economic indicators for investigated farms are depicted in Table 1. The average number of dairy cows per farm was 14 varying between 7 and 166 animals among investigated farms. According to the welfare protocol for dairy cows used for the ClassyFarm system the average biosecurity score for farms was 19.24 out of 30, while the welfare score averaged 57.73 out of 81 (Table 1). The average milk production was 7269 kg per cow and year with 4.04% fat and 3.38% protein (Table 1). The SCS averaged at 3.61. Finally, the average milk price calculated with respective milk composition (fat, protein, SCC) according to the pricing system of the cooperative Bergmilch South Tyrol was 0.53 eurocent per kg milk (Table 1).

**Table 1:** Descriptive summary of herd size, biosecurity score, welfare scores, production traits, and economic indicators across 2291 dairy farms.

Variable	Definition	Min	Max	Mean	Std	(25%) Q1*	(75%) Q3*
Herd size (number of cows)	Number of dairy cows (milking and dry)	7	166	14	6.1	8	21
Biosecurity score (30 scores)	Biosecurity practices in all the farms (n = 2291)	6.75	24	19.24	1.95	18	20.25
Welfare score (81 scores)	Total welfare (Area A, B, and C) n = 2291 farms	41	70	57.73	4.05	55	60
Area A scores (27 scores)	Area A assesses various aspects of animal welfare, including staffing, expertise, cleanliness, nutrition, health monitoring, and disease prevention	15	26	19.98	1.92	19	21
Area B scores (31 scores)	Area B evaluates critical factors related to the housing and environmental conditions for animals. These include the design and features of the housing system, space availability for lying and feeding, flooring, equipment for animal handling, provisions for calves of different ages, facilities for sick animals, and environmental parameters such as temperature, humidity, ventilation, and gas concentrations	10	26	18.77	2.78	17	20
Area C scores (23 scores)	Area C focuses on the health and physical condition of the animals. These scores encompass various aspects such as behavioral responses (human avoidance test), body condition score, cleanliness, skin health, lameness, claw condition, udder health, mastitis treatments, mortality rate, mutilations (e.g., disbudding, dehorning, tail docking), dystocia rate, and behavior changes	11	23	18.9	1.88	18	20
Milk production and quality traits							
SCS	Somatic cell score: SCS = log 2(SCC/100) + 3	1.31	7.5	3.61	0.89	3.05	4.22
Milk production (kg/year) per cow	The quantity of milk produced by an individual cow in kilograms per year	2498	12,712	7269	1588	6122	8352
Fat (%)		0.345	6	4.04	0.35	3.83	4.23
Protein (%)		2.69	4.13	3.38	0.18	3.27	3.5
Fat to protein ratio	Indicator in dairy farming as it provides insights into the nutritional composition of milk	0.10	1.79	1.20	0.09	1.14	1.25
Economic indicator							
Milk price (Euros per kg milk)	Milk prices are calculated considering respective milk composition (fat, protein, SCC) according to the pricing scheme of the cooperative Bergmilch South Tyrol	0.336	0.661	0.529	0.027	0.5	0.548

*Q1 (First Quartile): This is the median of the lower half of the data set. It represents the point below which 25% of the data falls; Q3: This is the median of the upper half of the data set. It represents the point below which 75% of the data falls.

### Interaction between welfare and biosecurity indices with milking performance and quality

Pearson correlations reported in Table 2 reveal a strong association between fat and protein with both, the animal welfare (0.74 and 0.68, respectively) and the biosecurity score (0.79 and 0.84, respectively). Furthermore, milk price strongly correlated with welfare and biosecurity score of a farm. The latter is explainable by the fact that the main parameter influencing the milk price is fat followed by protein as shown in Table 2. Furthermore, a moderate association was observed for milk production and welfare and biosecurity score (0.56 and 0.37, respectively).

**Table 2:** Pearson correlations between the average score of total animal welfare score, biosecurity scores, milk price, milk production, SCS, and quality traits across 2291 farms included in the study. (P < 0.05).

Variable	Welfare	Biosecurity	SCS	Fat, %	Protein, %	Milk production	Milk price €/kg milk
Welfare	1						
Biosecurity	0.05	1					
SCS	− 0.16	− 0.14	1				
Fat, %	0.74	0.68	0.00	1			
Protein, %	0.79	0.84	0.13	0.39	1		
Milk production	0.56	0.37	0.05	0.17	0.24	1	
Milk price €/kg milk	0.90	0.85	-0.27	0.93	0.61	0.19	1

### Effect of biosecurity and welfare on farm economy

**Figure 1:**
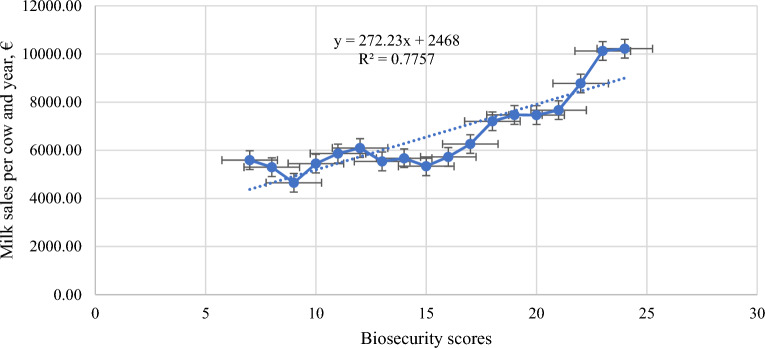
Association between Biosecurity Score and milk sales per cow and year.

**Figure 2:**
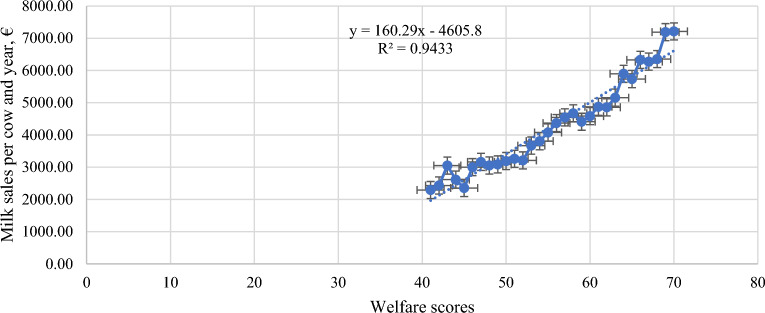
Association between Welfare Score and milk sales per cow and year.

Tables 3 and 4 depicts Least Square Means of biosecurity and welfare score as well as ECM, related milk price and predicted profitability per cow and year. For both biosecurity and welfare an increase in respective index resulted in an increase of predicted profitability per cow and year. In terms of numbers an increase of one score for biosecurity resulted in an increase in sales of 272.23 € per cow and year (R^2^ = 0.78) (Fig. 1) and increase of one score for welfare resulted in an increase in sales of 160.29 € per cow and year (R^2^ = 0.94) (Fig. 2). This information was considered for calculating estimates reported in Table 5 for quantifying the actual increase in farm profitability when improving welfare and biosecurity status. A significant increase in ECM and a significant decrease in SCS was observed with increasing welfare and biosecurity indices resulting in a significant increased milk price in farms corresponding to the highest class of biosecurity and welfare score (Table 5). For instance, farms with a biosecurity index score of 20 to 30 earned, on average, approximately 0.1 euro more per kg sold milk than farms having a biosecurity index below 10 (Table 5). In terms of animal welfare, farms with a higher welfare score (61 to 81 index points) earned 0.17 euro more per kg sold milk than farms having a low welfare score (< 45 index points) (Table 5).

**Table 3:** Least squares mean (LSM) and confidence limits of the mean (CLM) of the average ECM and SCS and the biosecurity score index, and the profitability per kg/year per cow.

Biosecurity scores						
Score index (total = 30)	Milk production (kg cow per year)			Milk price €/kg milk	Profitability per kg/year in Euros	Milk sales per cow and year (€)
	ECM	95% confidence limits				
7	5589.00	2828.79	8349.21	0.49	5589.49	4373.24
8	5295.00	3701.39	6888.61	0.50	5295.50	4645.45
9	4647.50	3520.65	5774.35	0.51	4648.01	4917.66
10	5444.83	4317.98	6571.68	0.54	5445.37	5189.87
11	5862.67	5212.08	6513.25	0.54	5863.21	5462.08
12	6092.17	4965.32	7219.02	0.55	6092.72	5734.29
13	5535.36	4492.10	6578.62	0.56	5535.91	6006.5
14	5665.81	4689.93	6641.69	0.57	5666.38	6278.71
15	5336.35	4463.49	6209.21	0.61	5336.96	6550.92
16	5723.62	5054.17	6393.07	0.57	5724.19	6823.13
17	6258.12	6052.39	6463.85	0.60	6258.72	7095.34
18	7201.40	7046.86	7355.94	0.58	7201.98	7367.55
19	7466.20	7327.84	7604.56	0.60	7466.80	7639.76
20	7461.20	7366.70	7555.71	0.61	7461.81	7911.97
21	7665.14	7489.51	7840.77	0.59	7665.73	8184.18
22	8775.92	8540.10	9011.74	0.59	8776.51	8456.39
23	10,126.00	9786.42	10,466.00	0.58	10,126.58	8728.6
24	10,220.00	9299.62	11,140.00	0.59	10,220.59	9000.81

**Table 4:** Least squares mean (LSM) and confidence limits of the mean (CLM) of the average ECM and SCS and the total welfare score index, and the profitability per kg/year per cow.

Welfare scores						
Score index (total = 81)	Milk production (kg cow per year)			Milk price €/kg milk	Profitability per kg/year in Euros	Predictive milk sales per kg/year in Euros
	ECM	95% confidence limits				
41	4495.00	2772.80	6217.20	0.51	2292.45	1966.14
42	4529.00	2806.80	6251.20	0.54	2432.86	2126.43
43	5485.00	3049.44	7920.56	0.56	3051.20	2286.72
44	4732.00	3325.83	6138.17	0.55	2613.84	2447.01
45	4068.83	3074.52	5063.15	0.58	2354.32	2607.30
46	5348.25	4130.47	6566.03	0.56	3000.78	2767.59
47	5934.50	5164.31	6704.69	0.53	3163.44	2927.88
48	5429.50	4838.79	6020.21	0.56	3054.31	3088.17
49	5254.70	4735.44	5773.97	0.59	3086.04	3248.46
50	5451.21	5061.20	5841.21	0.59	3190.29	3408.75
51	5551.10	5260.00	5842.21	0.59	3260.76	3569.04
52	5707.36	5375.92	6038.80	0.56	3211.37	3729.33
53	6349.03	6095.10	6602.95	0.58	3669.78	3889.62
54	6453.85	6222.68	6685.03	0.59	3806.62	4049.91
55	6729.43	6542.08	6916.78	0.61	4076.86	4210.20
56	7388.08	7217.56	7558.60	0.59	4367.39	4370.49
57	7611.82	7449.09	7774.55	0.60	4544.87	4530.78
58	7426.07	7272.64	7579.49	0.63	4669.70	4691.07
59	7283.25	7119.79	7446.72	0.61	4411.82	4851.36
60	7745.76	7588.21	7903.30	0.59	4583.01	5011.65
61	8063.16	7872.98	8253.35	0.60	4872.25	5171.94
62	8100.33	7898.07	8302.59	0.60	4855.91	5332.23
63	8469.27	8212.54	8726.00	0.61	5156.43	5492.52
64	9381.59	9058.99	9704.18	0.63	5896.98	5652.81
65	9569.85	9179.84	9959.85	0.60	5733.96	5813.10
66	10,301.00	9906.17	10,696.00	0.61	6327.90	5973.39
67	10,468.00	9606.65	11,329.00	0.60	6274.00	6133.68
68	10,170.00	8952.64	11,388.00	0.62	6353.20	6293.97
69	11,277.00	9554.80	12,999.00	0.64	7188.97	6454.26
70	11,458.00	9022.44	13,894.00	0.63	7212.01	6614.55

**Table 5:** Estimate and standard error (SE) of the ECM, SCS, and milk price for the effect of biosecurity, total welfare, and emergency management scores in 2291 dairy farms; *SD* standard deviation.

Independent variables	Score index	ECM			SCS			Milk price €/kg milk		
		Estimated coefficients (β)	SE	P-value	Estimated coefficients (β)	SE	P-value	Estimated coefficients (β)	SE	P-value
Intercept		7351.24	561.8	< 0.0001	3.45	0.18	< 0.001	0.516	0.016	< 0.001
Biosecurity score	< 10	Ref.			Ref.			Ref.		
	10 to 15	337.6	15.54	< 0.0001	− 0.009	0.0021	< 0.0001	0.0053	0.0004	na
	16 to 20	381.9	15.84	< 0.005	− 0.027	0.0093	< 0.005	0.012	0.0015	na
	20 to30	426.3	22.72	< 0.0001	− 0.098	0.0406	< 0.018	0.092	0.0186	< 0.025
Total welfare score	< 45	Ref.			Ref.			Ref.		
	45 to 60	235.34	6.57	< 0.001	− 0.0563	0.0045	< 0.002	0.027	0.0017	< 0.012
	61 to 81	419.98	37.16	< 0.035	− 0.063	0.0091	< 0.001	0.169	0.0038	< 0.019

Irrespective of this, our results also evidence a poor to moderate adoption of biosecurity measures in mountain dairy farms as most of investigated farms had no or moderate knowledge about certain biosecurity measures like udder health analysis, control plans for infectious diseases or preventive control of endo and ectoparasites (Table 6). In contrast, adequate animal welfare measures were found in most of the farms in the different sections regarding farm management and staff training (Area A), housing (Area B) and animal-based measures (Area C) (Table 7).

**Table 6:** Estimate and standard error (SE) of the MP for the effect of biosecurity measures scores with a significant impact on milk production across 2291 dairy farms.

Independent variables	Score index		N of farms	Percent %	ECM (kg per cow/year)			Economic index	
					Estimated coefficients (β)	SE	P-value		
Biosecurity practice (measure)								ECM (kg per cow/year)	Milk sales per cow and year (€)
Intercept					7144.86	486.95	< 0.001		
Control and prevention of infectious diseases	0	No knowledge of the most important infectious diseases	508	22.18	Ref.			Ref.	Ref = 3779.63
ECM = 7144.86 + 261.7 × 1	1	Partial knowledge of the most important Infectious diseases and not defined plans	1710	74.62	261.7	119.85	< 0.031	7405.7	3917.615
ECM = 7144.86 + 127.98 × 2	2	Knowledge about the 3 most important infectious diseases	73	3.20	127.89	152.13	na	7399.78	3914.484
Submission and examination of pathological/biological	0	No (in the last 12 months)	362	15.80	Ref.				
ECM = 7144.86 + 19.77 × 2	2	Yes (in the last 12 months)	1929	84.20	19.77	17.62	< 0.048	7183.54	3800.093
Udder health analyses	0	No analysis	1650	72.00	Ref.				
ECM = 7144.86 + 226.18 × 1	1	Collective analysis of the whole stock	217	9.46	226.18	114.83	< 0.033	7370.18	3898.825
ECM = 7144.86 + 311.74 × 2	2	Analysis of problem animals available	425	18.54	311.74	270.39	< 0.044	7767.48	4108.997
Preventive control of endo ectoparasites	0	No knowledge or no plans	233	10.16	Ref.				
ECM = 7144.86 + 7.42 × 1	1	Partial knowledge and/or not defined prevention plans	1939	84.63	7.42	16.85	na	7151.42	3783.101
ECM = 7144.86 + 25.557 × 2	2	Good knowledge and programmed antiparasitic Treatments	119	5.21	25.557	22.13	< 0.009	7195.114	3806.215
Control and analysis of the used water	0	Analysis not available when using water from deep well	553	24.12	Ref.				
ECM = 7144.86 + 43.68 × 2	2	Use of municipal water/annual analysis present	1738	75.88	43.68	31.46	< 0.007	7231.36	3825.389

**Table 7:** Estimate and standard error (SE) of the MP for the effect of welfare measures scores with a significant impact on milk production across 2291 dairy farms and the economic index.

Independent variables	Score index		N of farms	Percent %	ECM			Economic index	
					Estimated Coefficients (β)	SE	P-value		
Total welfare								ECM (kg per cow/year)	Milk sales per cow and year (€)
Intercept					7787.36	522.8	< 0.033	Ref = 7787.36	Ref = 4119.51
Area A—farm management and staff training									
Number of inspections of the animals: (cattle > 6 months old—calves)	0	< 1 inspection/day	40	1.75	Ref.			Ref.	
	1	1 or more than 1 inspection/day	2251	98.25	234.46	112.64	< 0.015	8021.82	4243.54
Treatment of sick or injured animals	0	Not adequate: evidence of untrained staff and/or presence of animals in need of treatment but haven’t received it yet	0	0.00	Ref.				
	1	Adequate: presence of trained personnel with evidence of animals in the infirmary (area, box, or station) or undergoing treatments	2019	88.13	80.46	10.24	< 0.0002	7867.82	4162.08
	2	Optimal: in addition to the criteria for adequacy, the presence of written procedures for the treatment of animals (e.g., mastitis management plan, lameness plan, or specific therapeutic protocols)	272	11.87	216.57	142.75	< 0.044	8220.5	4348.64
Management of feed and daily ration	0	Not adequate	15	0.65	Ref.				
	1	Adequate	1746	76.21	− 28.3	8.95	na	7759.06	4104.54
	2	Optimal: the presence of an optimal feed for the animals, calculated by a nutritionist, frequently reviewed, or revised with every change of feed, and composed of healthy food	530	23.13	48.4	16.44	< 0.0043	7884.16	4170.72
Feed availability	0	Feed not available for 24 h	30	1.31	Ref.				
	1	Separate ration correctly managed (concentrates administered at least 2 times/day) and available for 24 h	1577	68.83	124.34	11.5	< 0.007	7911.7	4185.29
	2	Total mixed ration available for 24 h	684	29.86	347.8	191.7	< 0.049	8482.96	4487.49
Water provision	0	1 or more animals without access to drinking water, GRAZING: excessive distances between water sources and grazing areas	186	8.12	Ref.				
	1	All animals have access to functioning water troughs, GRAZING: presence of artificial watering troughs or natural water sources positioned near grazing areas	1407	61.41	24.34	59.24	< 0.006	7811.7	4132.39
	2	Presence of functioning “level” water troughs in all groups and ad libitum water for all calves, including those in cages	698	30.47	330.1	81.35	< 0.0091	8447.56	4468.76
Cleanliness of water points	0	Both drinkers and water are dirty	33	1.44	Ref.				
	1	There are feed residues in the drinkers but water is fresh and clean	1808	78.92	213.5	149.86	< 0.0026	8000.86	4232.45
	2	Both drinkers and water are clean	450	19.64	415.7	219.8	< 0.0075	8618.76	4559.32
Prevention of hoof disorders	0	Absence of hoof trimming plans and no possibility of hoof baths	27	1.18	Ref.				
	1	Annual hoof trimming plans and periodic hoof baths carried out randomly	2058	89.83	10.9	11.6	< 0.0058	7798.26	4125.28
	2	Semi-annual hoof trimming plans and weekly hoof baths carried out regularly	206	8.99	392.7	227.65	< 0.038	8572.76	4534.99
Hygiene of milking areas and equipment	1	Considered adequate: proper cleaning of the groups, but feces on floors and walls are present	1000	43.65	Ref.				
	2	Considered optimal: absence of feces and good overall hygiene	1291	56.35	77.22	68.39	< 0.026	7941.8	4201.21
Management of milking operations and udder hygiene	0	Not adequate: absence of udder hygiene or failure to observe oxytocin times or incorrect use of the milking machine and improper attachment and detachment of the clusters	41	1.79	Ref.				
	1	Considered adequate: proper udder cleaning and observation of oxytocin times/hand milking of the udder and correct management of milking operations and equipment	1009	44.04	51.92	62.67	< 0.017	7839.28	4146.98
	2	Considered optimal: proper udder cleaning, adherence to oxytocin times, and use of pre/post dipping with spray or clean cups/hand milking with excellent disinfection	1241	54.17	122.14	74.26	< 0.0057	8031.64	4248.74
Area B—housing									
Available space at feed bunk	0	In case of separate ration, number of feeding places < 100% of the total number of animals, or in case of total mixed ration, number of feeding places < 70% of the total number of animals	60	2.62	Ref.				
	1	In case of separate ration, as many feeding places as animals, or in case of total mixed ration, number of feeding places ≥ 70% of the total number of animals	624	27.24	226.29	66.886	< 0.0007	8013.65	4239.22
	2	STALL: presence of 2 differentiated access points, with a total number of spaces greater (by more than 20%) than the number of animals, or the possibility of access to suitable grazing (even periodically, for 60 days per year).GRAZING: availability of ample grazing areas and/or presence of adequate integration points according to the number of animals	1607	70.14	− 47.16	12.79	na	7693.04	4069.62
Functioning and number of water points	0	Less than 1 functioning water bowl for 10 animals or less than 6 cm of trough per animal	852	37.19	Ref.				
	1	1 functioning water bowl for 10 animals or 6 cm of trough per animal	755	32.96	38.63	42.6	< 0.043	7825.99	4139.95
	2	More than 1 functioning water bowl for 10 animals or more than 6 cm of trough per animal, and different water access points	684	29.86	50.45	33.68	< 0.002	7888.26	4172.89
Facilities for sick animals	0	Lack of facilities for sick animals or presence of sick pens with fully slatted floors (no bedding)	1595	69.62	Ref.				
	1	Presence of facilities for sick animals provided with deep litter	642	28.02	119.46	56.12	< 0.022	7906.82	4182.71
	2	Presence of specific facilities for sick animals that prevent contact with healthy animals and provided with clean deep litter	54	2.36	− 31.57	11.36	na	7724.22	4086.11
Temperature, humidity, and ventilation	0	Inadequate temperature and humidity, or insufficient air movement, or closed buildings	43	1.88	Ref.				
	1	Adequate temperature and humidity and sufficient natural air movement or ventilation, but ventilation does not have an automatic control system	1820	79.44	80.6	95.66	na	7867.96	4162.15
	2	Adequate temperature and humidity, thanks to an automatic ventilation or cooling system; otherwise, summer pasture equipped with shelters	428	18.68	211.43	93.14	< 0.007	8210.22	4343.21
Area C—animal-based measures									
Human avoidance test	0	Animals cannot be approached as close as 100 cm	17	0.74	Ref.				
	1	Animals can be approached as close as 100 to 50 cm but cannot be touched	310	13.53	90.91	22.19	< 0.044	7878.27	4167.60
	2	Animals can be approached closer than 50 cm and can be touched	1964	85.73	116.53	85.6	< 0.0005	8020.42	4242.80
Body condition score (BCS)	0	> than 10% of the animals are very lean (BCS ≤ 2)	78	3.40	Ref.				
	1	5–10% of the animals are very lean (BCS ≤ 2)	235	10.26	118.17	73.89	< 0.016	7905.53	4182.03
	2	< Than 5% of the animals are very lean (BCS ≤ 2)	1978	86.34	341.78	184.16	< 0.0147	8470.92	4481.12
Cleanliness of the animals	0	> Than 20% of dirty animals	576	25.14	Ref.				
	1	10–20% of dirty animals	457	19.95	10.19	4.52	< 0.006	7797.55	4124.90
	2	< Than 10% of dirty animals	1258	54.91	111.28	74.61	< 0.0449	8009.92	4237.25
Skin alterations	0	> Than 30% of the animals with integument alterations	23	1.00	Ref.				
	1	15–30% of the animals with integument alterations	267	11.65	228.69	113.7	< 0.008	8016.05	4240.49
	2	< Than 15% of the animals with integument alterations	2001	87.34	410.86	205.73	< 0.0395	8609.08	4554.20
Lameness	0	> Than 8% of lame animals	174	7.59	Ref.				
	1	4–8% of lame animals	219	9.56	122.8	84.7	< 0.038	7910.16	4184.47
	2	< Than 4% of lame animals	1898	82.85	315.93	258.32	< 0.007	8419.22	4453.77
Prevalence of long and deformed claws	0	More than 40% of animals with long and deformed claws	40	1.75	Ref.				
	1	Between 10 and 40% of animals with long and deformed claws	253	11.04	14.6	16.14	na	7801.96	4127.24
	2	Less than 10% of animals with long and deformed claws	1998	87.21	153.94	85.64	< 0.048	8095.24	4282.38
Evaluation udder health	0	Somatic cell geometric mean > 400.000 cell/ml	11	0.48	Ref.				
	1	Somatic cell geometric mean between 300.000 and 400.000 cell/ml	2280	99.52	288.96	172.45	< 0.008	8076.32	4272.37
Number of treatments for clinical mastitis in 1 year	0	Number of treatments exceeding 80% of the number of lactating cows or inability to retrieve the data	0	0.00	Ref.				
	1	Number of treatments ranging from 40 to 80% of the number of lactating cows	86	3.75	− 84.87	24.19	na	7702.49	4074.62
	2	Number of treatments less than 40% of the number of lactating cows	2205	96.25	183.48	109.39	< 0.0064	8154.32	4313.64

## Discussion

To the best of the authors’ knowledge, this study represents the first comprehensive investigation into the association between animal welfare, biosecurity (estimated based on the welfare protocol for dairy cows of the ClassyFarm system), milk quality, and milk performance on a large scale, encompassing 2291 dairy farms in mountainous regions. Therefore, observed results offer a detailed and realistic understanding of how biosecurity and welfare measures interact with milk performance (both quality and yield) and subsequently influence economic performance.

Overall, our results reveal a poor to moderate adoption of biosecurity measures (19.24 out of 30, as shown in Table 1) in mountain dairy farms. This deficiency can be partly attributed to structural limitations in farm buildings due to topographic disadvantages in such marginal areas, as highlighted by Holighaus et al.^20^, and partly to farmers’ lack of awareness, as indicated in our results (see Table 6). This aligns with previous studies by Correia-Gomes et al.^21^ and Van Steenwinkel et al.^22^ which reported limited awareness of biosecurity practices and poorer farm infrastructure (such as animal housing) in small-scale farms compared to larger operations. Additionally, farmers often perceive certain biosecurity measures as impractical due to the economic and logistical burdens associated with their implementation in day-to-day operations^15,23^. Irrespective of this, previous studies such as that by Robertson^24^ highlight the importance of adopting biosecurity measures to safeguard animal health, which is crucial for reducing the need for antimicrobial substances in the livestock sector. Specifically, the adoption of biosecurity measures and plans is essential for maintaining disease-free farms, regions, and countries, thereby contributing positively to the reduction of antimicrobial resistance in the livestock sector^5^. Moreover, studies emphasize the importance of appropriate education, as well as farmer’s knowledge and attitudes, in enhancing the adoption of biosecurity measures to minimize disease spread and improve farm productivity^14,25,26^. In this context, Shortall et al.^23^ suggest that consulting veterinarians can play a pivotal role in advising farmers on biosecurity practices. Furthermore, the adoption of biosecurity practices has been shown to have a significant impact on the economic outcomes of dairy farms^17^. Hereby, Osawe et al.^17^ named good managerial abilities to be crucial for implementing biosecurity measures effectively and thus ensuring good economic viability of the farm. The economic significance of properly adopting biosecurity measures is evident in our results (Tables 3, 5, Fig. 1), providing additional motivation for farmers to improve the current situation. Nevertheless, the peculiarity of small-scale mountain dairy farming needs to be considered by the EU policy to adapt certain biosecurity measures to those specific production conditions as already highlighted in Holighaus et al.^20^, to ensure the continued existence of those farms. The latter provides several crucial ecosystem services such as the conservation of diverse ecosystems and diverse flora and fauna including rare species that depend on traditional mountain agricultural practices (e.g. transhumance) as well as the maintenance of terraced fields, grazing lands, and mountain meadows help to prevent soil erosion^27^. Moreover, they will increasingly contribute to global food security as they mostly use human-inedible grassland through ruminant production systems for producing food (e.g.^28^).

In terms of animal welfare, our results revealed a moderate to good welfare status in the farms assessed (57.73 out of 81, as shown in Table 1). This underscores farmers’ existing awareness of the importance of maintaining high welfare standards for their dairy cows to ensure good herd productivity. Animal welfare, among other factors, is significantly associated with the economic success of a farm as shown in Table 2. Indeed, our results demonstrated an increase in sales with improved animal welfare (Tables 4, 5). This finding aligns with Villettaz Robichaud et al.^29^, who demonstrated the positive impact of good comfort and welfare on dairy herd productivity. Conversely, Coignard et al.^30^ found that while higher milk production may occur, it does not necessarily reflect the overall welfare status of a herd, as it can be linked to an increased occurrence of health disorders like metabolic diseases. Therefore, Beck and Gregorini^31^ emphasized the importance of ensuring livestock well-being by allowing animals to express natural behaviors fully in a domesticated environment, leading to a positive emotional experience and improved welfare and health for dairy cows. Additionally, the smaller scale of mountain dairy farms (Table 1) may positively influence welfare due to potentially better individual animal care and human-animal bonding compared to large dairy enterprises. However, Lindena and Hess^32^ concluded from their study of 3085 dairy farms in Germany that farm size had only a limited effect on animal welfare, while farmers’ knowledge and skills in farm management played a more significant role. Similarly, Gieseke et al.^33^ argued that farm size alone cannot serve as an indicator of animal welfare, with housing conditions and management practices having a more substantial impact. Despite encouraging findings regarding animal welfare (Table 1), the prevalent housing system in the mountain area is still tie stalls^20,34^ for which several studies in the past^35^ as well as EFSA^36^ highlighted some deficiencies regarding animal health and welfare, especially when housed on a year-round basis^37^. Therefore, there remains significant room for improvement in enhancing animal welfare in small-scale mountain dairy farming, especially considering the increasing consumer awareness of animal welfare issues and ethical concerns surrounding animal production. Napolitano et al.^38^ emphasized the importance of providing consumers with accurate information about animal welfare-friendly production methods through effective labeling and scientifically validated monitoring systems to encourage willingness to pay more for animal-based products. However, farmers face the challenge that higher production costs to ensure better welfare standards are often not offset by higher market prices, as many consumers remain indifferent to animal welfare when purchasing animal-based products^39^. Nonetheless, maintaining good animal welfare standards is crucial not only for the economic success of farms, as shown in our results (Tables 4, 5, Fig. 2), but also for ensuring social acceptance of livestock production.

## Conclusions

The results of the present study provide comprehensive insights into the interaction between animal welfare, biosecurity, milk quality, and milk performance on 2291 dairy farms in mountainous regions. The findings highlight the importance of both biosecurity and animal welfare measures in enhancing productivity and economic performance. Nevertheless, our results reveal a poor to moderate adoption of biosecurity measures in mountain dairy farms, attributed partly to structural limitations of small-scale mountain farms and farmers’ lack of awareness. This aligns with previous research highlighting the need for increased education and support for farmers to effectively implement biosecurity practices. In contrast, the study indicates a moderate to good welfare status in the assessed farms, reflecting farmers’ awareness of the importance of maintaining high welfare standards for dairy cows. Improved animal welfare is shown to have a positive impact on milk sales and productivity, highlighting the economic benefits of prioritizing animal well-being. However, there remains room for improvement, particularly in addressing animal housing deficiencies (e.g. year-round tie stall housing) and general consumer concerns regarding animal welfare in livestock production systems. Overall, the study underscores the need for continued efforts to promote and support the adoption of biosecurity measures while simultaneously enhancing animal welfare standards. However, the peculiarity of mountain livestock farming and its crucial role for the provision of some important ecosystem service needs to be considered by the policymakers when developing regulations regarding biosecurity and animal welfare for ensuring its continued existence. Moving forward, targeted interventions and educational initiatives should be prioritized to empower farmers and promote best practices in biosecurity and animal welfare management. However, the resultant increase in production costs necessitates society's willingness to pay more for animal-based food.

## Methods

For the present study, 3469 dairy farms all located in the province of South Tyrol (very northern part of Italy) were visited and assessed in 2022 by specifically trained auditors with the welfare protocol for dairy cows used in the ClassyFarm system. The specific details of the assessment and the protocol as well as farm structure and management are published in Holighaus et al.^20^. For the assessment only dairy cows were considered. In assessing the biosecurity measures and animal welfare criteria within our study, we adopted a structured approach to quantify their prevalence across different farms. Each question related to biosecurity measures or welfare criteria was assigned a numerical value of 0, 1, or 2, indicating the absence, partial absence, or total presence of the measure or criteria, respectively. By summing the scores assigned to each relevant question, we computed a cumulative score for both biosecurity measures and welfare criteria for each farm included in our study. This method enabled us to quantify the level of biosecurity and welfare and to determine the percentage of farms that exhibited specific measures or criteria. Furthermore, milk samples from farms that permitted us were collected every 5 weeks in the course of the official milk performance control and subsequently analyzed for the classical milk quality traits (milk fat, milk protein) and somatic cell count (SCC) in the laboratories of the South Tyrolean Dairy Association equipped with MilkoScan FT6000 (Foss, Hillerød, Denmark) and Fossomatic FC (Foss, Hillerød, Denmark). Fat-to-protein ratio (FPR) was calculated as FPR = FP/PP. For milk production (MP), fat percentage in milk (FP), protein percentage in milk (PP), FPR, and lactose percentage (LP), values were retained only if they fell within the range of mean ± 3 standard deviations (SD). For SCC, the minimum value was set at 1000 cells/mL, while the maximum value was set at 10,000,000 cells/mL. To achieve a normal distribution, SCC was transformed into somatic cell score (SCS) using the conventional formula proposed by Ref.^40^. Moreover, the milk pricing system for conventional milk of the cooperative Bergmilch Südtirol was used for formulating the milk price for respective milk quality as most of the South Tyrolean dairy farms are members of this cooperative. The pricing systems considers fat, protein, and somatic cells in milk. The detailed description of the payment system is provided in Supplementary Material 1. Additional payments in the milk pricing system for animal welfare is currently not foreseen in the pricing system. Furthermore, natural changes in the milk price on the market were not considered in the economic evaluation. The final dataset used for the statistical analysis comprised milk and biosecurity data from 2291 dairy farms.

### Statistical analysis

The data were analyzed using SAS 9.4 (SAS Institute Inc., Cary, NC, USA), treating each farm as an experimental unit. Normality assumptions were assessed using multiple methods, including the Shapiro–Wilk test, examination of skewness and kurtosis, and visual inspection of normal probability plots. Descriptive statistics, including mean, standard deviation, minimum, maximum, and quartiles, were computed using PROC MEANS efficiently summarizing the dataset’s central tendencies and distribution characteristics. Pearson correlation coefficients were derived via PROC CORR and used to investigate the relationships between Welfare and Biosecurity scores, SCC, Fat, %, Protein, %, Milk production, and Milk Price. Results are presented as the correlation coefficient (Rho). Subsequently, the impact of biosecurity and welfare scores on various economic indicators, such as milk production, and profitability in euros, was examined using PROC REG, enabling the evaluation of linear relationships and the estimation of regression coefficients, standard errors, and *P-*values. Additionally, the robustness of the results was tested using bootstrapping techniques. Only the biosecurity and welfare indicators having a significant impact on milk production and consequently, economic indices were used for subsequent analysis. An ANOVA test in the GLM procedure of SAS was used to check for comparing biosecurity and animal welfare scores with milk production, milk price, and profitability. Results are presented as least squares mean (LSM) standard error of the mean (SEM). This analysis unveiled significant associations between Biosecurity and Welfare scores and economic performance metrics. Moreover, independent variables related to Biosecurity and Welfare practices were scrutinized using PROC REG, elucidating their impact on economic indicators such as ECM (kg per cow/year), and Milk sales per cow and year (€). These findings underscored the significance of specific practices, including control and prevention of infectious diseases, Submission and examination of pathological/biological material, udder health analyses, preventive control of endo ectoparasites, control and analysis of the used water, number of inspections of the animals, treatment of sick or injured animals, management of feed and daily ration, feed availability, water provision, cleanliness of water points, prevention of hoof disorders, hygiene of milking areas and equipment, management of milking operations and udder hygiene, available space at the feed bunk, functioning and number of water points, facilities for sick animals, temperature, humidity, and ventilation, human avoidance test, body condition score (BCS), cleanliness of the animals, skin alterations, lameness, prevalence of long and deformed claws, evaluation udder health, and number of treatments for clinical mastitis in 1 year.

The equation used in the analysis typically follows the form of a linear regression model:

$$Y=\beta 0+\beta 1X1+\beta 2X2+\cdots +\beta nXn+\varepsilon ,$$

where Y represents the dependent variable. *X*1, *X*2,…, *Xn* represent the independent variables, which are specific measures related to welfare or biosecurity practices. *β*0, *β*1, *β*2,…, *βn* are the regression coefficients corresponding to each independent variable. *ε* represents the error term.

A Tukey–Kramer adjustment was used to account for multiple comparisons. The criterion for determination of statistical significance was established at P < 0.05.

### Ethics declarations

The experimental and notification procedures were carried out in compliance with Directive 86/609/EEC.

### Supplementary Information

Supplementary Information.

## Data Availability

The data that support the findings of this study are available on request from the corresponding author, [TZ]. The data are not publicly available due to restrictions.
