# Supplementary material for: Assessing the impact of biosecurity practices and animal welfare in small-scale mountain dairy farming
Source: Sci Rep. 2024 Jun 10;14:13294. doi: 10.1038/s41598-024-63841-y (PMC11164905; doi:10.1038/s41598-024-63841-y)
Supplement: Supplementary file 1 — Supplementary Information. [file 41598_2024_63841_MOESM1_ESM.docx]

SM1 Method for estimating the pricing system of dairy cooperatives in South Tyrol

Total revenue= 0.046€ per kg fat + 0.036 per kg protein

Bonus fat:

< 3.80 % fat                0 Eurocent

3.80 – 3.89 % fat        0.50 Eurocent

3.90 – 3.99 % fat        1.00 Eurocent

4.00 % fat                   1.50 Eurocent

Bonus/deduction somatic cells

| Somatic Cell count *1000 | Bonus/Deduction/ per kg Milk |
| --- | --- |
| <100 | 1.5 |
| 101-110 | 1.4 |
| 111-120 | 1.3 |
| 121-130 | 1.2 |
| 131-140 | 1.1 |
| 141-150 | 1.0 |
| 151-160 | 0.9 |
| 161-170 | 0.8 |
| 171-180 | 0.7 |
| 181-190 | 0.6 |
| 191-200 | 0.5 |
| 201-210 | 0.4 |
| 211-220 | 0.3 |
| 221-230 | 0.2 |
| 231-240 | 0.1 |
| 241-250 | 0.0 |
| 251-260 | -0.1 |
| 261-270 | -0.2 |
| 271-280 | -0.3 |
| 281-290 | -0.4 |
| 291-300 | -0.5 |
| 301-310 | -1.2 |
| 311-320 | -1.4 |
| 321-330 | -1.6 |
| 331-340 | -1.8 |
| 341-350 | -2.0 |
| 351-360 | -2.2 |
| 361-370 | -2.4 |
| 371-380 | -2.6 |
| 381-390 | -2.8 |
| 391-400 | -3.0 |
| 401-500 | -4.0 |
| 501-700 | -8.0 |
| >700 | -12.5 |
